# Rapid production of engineered human primary hepatocyte/fibroblast sheets

**DOI:** 10.1016/j.dib.2015.09.044

**Published:** 2015-10-09

**Authors:** Yusuke Sakai, Makiko Koike, Akihiko Soyama, Masaaki Hidaka, Tamotsu Kuroki, Susumu Eguchi

**Affiliations:** Department of Surgery, Nagasaki University Graduate School of Biomedical Sciences, 1-7-1 Sakamoto, Nagasaki 852-8501, Japan

**Keywords:** Human primary hepatocyte, Fibroblast, Cell sheet, Tissue engineering

## Abstract

This data article contains data related to the research article entitled “Vascularized subcutaneous human liver tissue from engineered hepatocyte/fibroblast sheets in mice,” published in *Biomaterials*[1]. Engineered hepatocyte/fibroblast sheets (EHFSs) are used for construction of vascularized subcutaneous liver tissue without a pre-transplant vascularization procedures. Here, we described a rapid production technique of EHFSs by controlling fibroblast density and coating fetal bovine serum (FBS) onto temperature-responsive culture dishes (TRCDs). The human fibroblast monolayer formed on FBS-coated TRCDs within 1 h when seeded at a high density (at least 1.56×10^5^ cells/cm^2^). The most rapid EHFS production was achieved soon after the adhesion of human primary hepatocytes onto the fibroblast layer.

**Specifications Table**

**Table:** 

Subject area	*Biology*
More specific subject area	*Tissue engineering, cell sheet, hepatocyte culture*
Type of data	*Image, graph, figure*
How data was acquired	*Microscope*
Data format	*Raw*
Experimental factors	*Cell sheet, rapid producing technique*
Experimental features	*Rapid production of engineered human hepatocyte/fibroblast sheet*
Data source location	*Nagasaki University Graduate School of Biomedical Sciences, Nagasaki, Japan*
Data accessibility	*Supplementary data of the article*

**Value of the data**

• FBS served as a good TRCD coating for the rapid preparation of fibroblast monolayers.
• Fibroblast monolayers formed within 1 h by seeding at least 1.56×10^5^ cells/cm^2^.
• Rapid production of EHFSs was achieved approximately 3 h after the first inoculation of TIG-118 cells.

## 1 Data and experimental design

### 1.1 Fibroblast monolayer preparation by controlling cell density and FBS-coating to TRCD

**Fig. 1:**
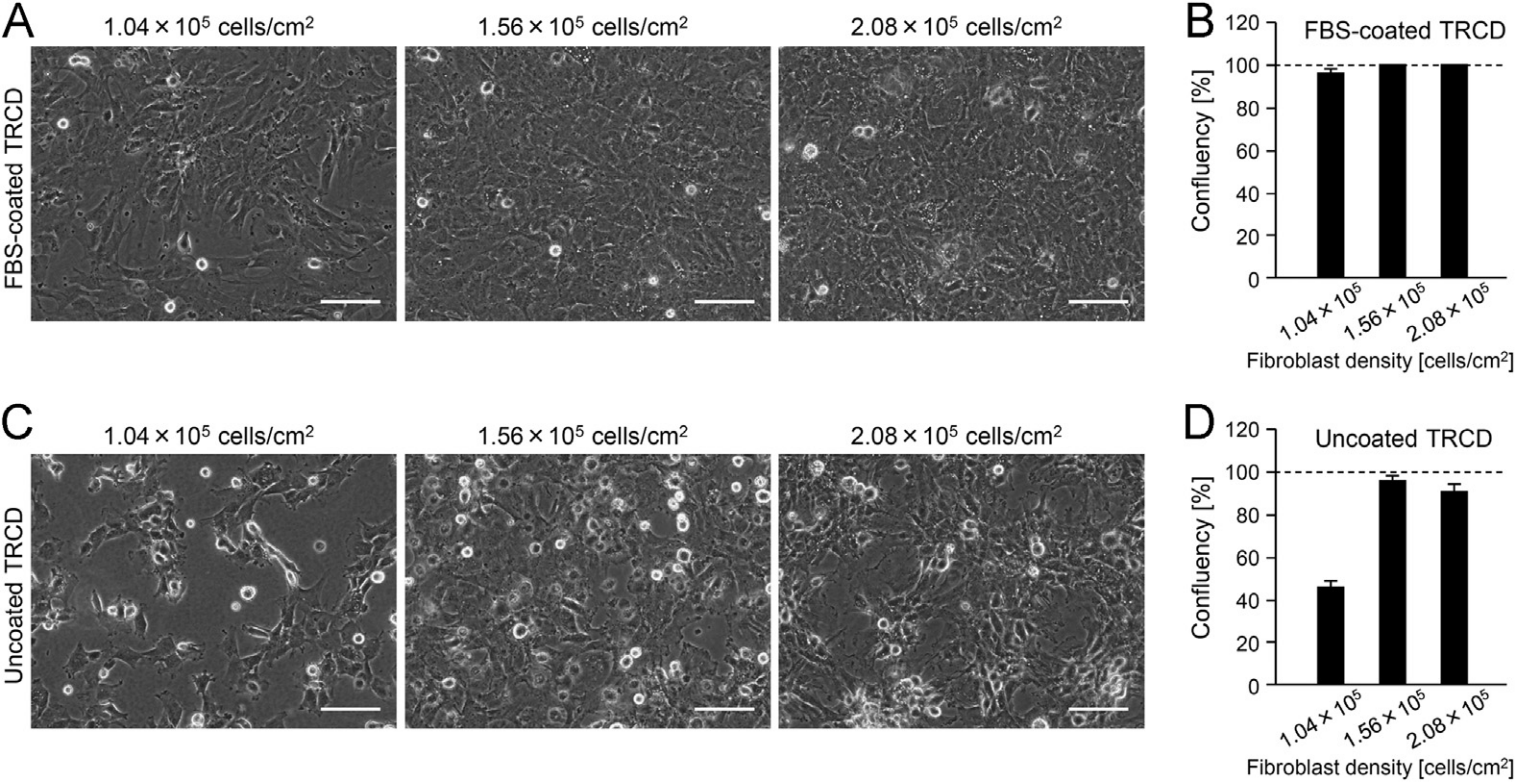
Phase-contrast micrographs (A, C) and confluency (B, D) of fibroblasts cultured on TRCDs at 2 h after inoculation. Fibroblasts were cultured at 1.04, 1.56, or 2.08×10^5^ cells/cm^2^ on (A, B) FBS-coated or (C, D) uncoated TRCDs. Scale bar, 100 μm. The dashed lines indicate the confluent.

Human fibroblasts (TIG-118 cells) formed a confluent monolayer within 1 h after inoculation with at least 1.56×10^5^ cells/cm^2^ onto FBS-coated TRCDs (Fig. 1A and B). Fibroblasts seeded at a lower density (1.04×10^5^ cells/cm^2^) did not form confluent monolayers. Fibroblasts on uncoated TRCDs were unable to reach confluence despite high-density inoculation and showed non-uniform cell distributions (Fig. 1C and D).

### 1.2 Human primary hepatocyte density for healthy culture on a FBS-coated TRCD

**Fig. 2:**
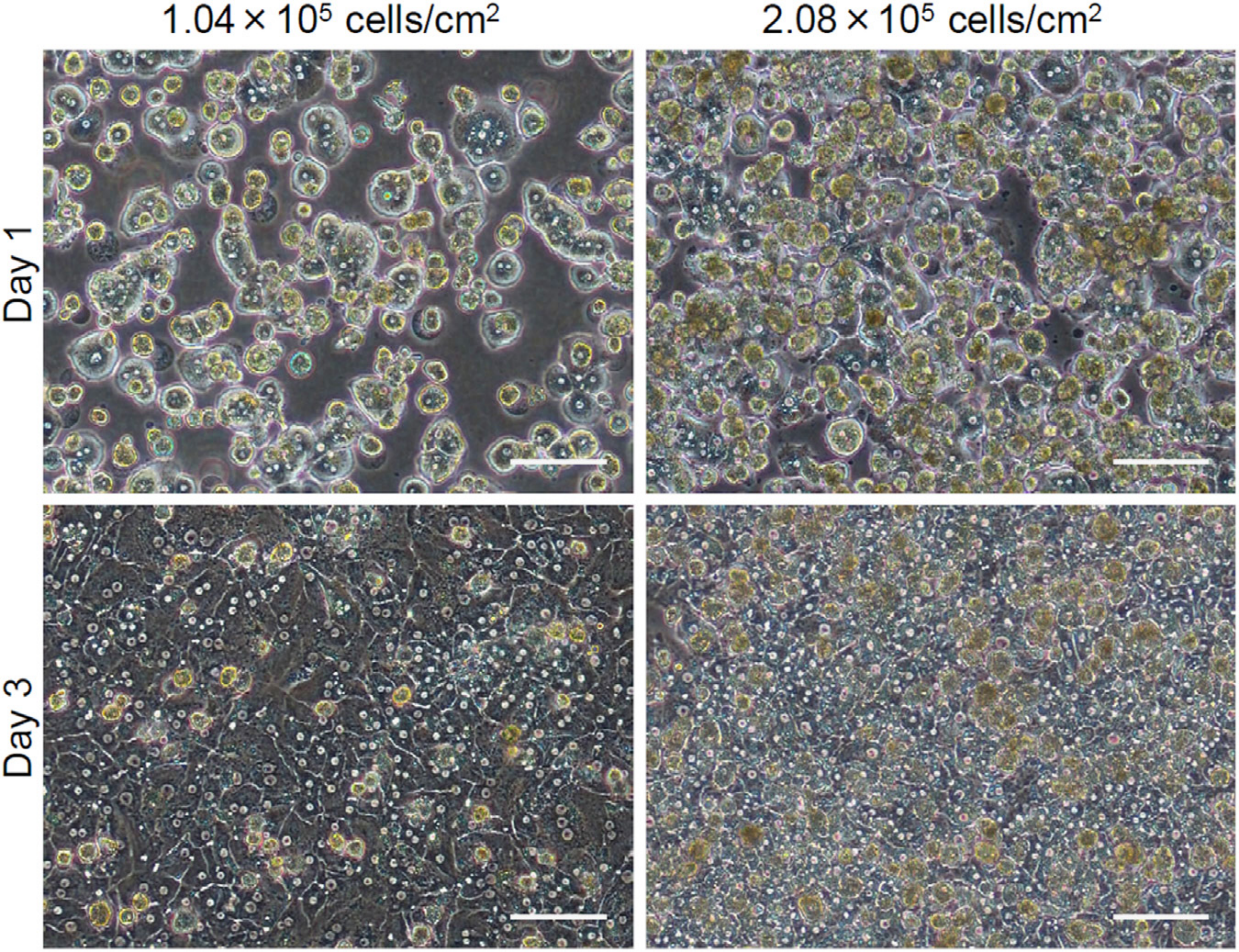
Phase-contrast micrographs of hepatocytes cultured on FBS-coated TRCDs at 1 and 3 days of culture. Hepatocytes were cultured at 1.04 or 2.08×10^5^ cells/cm^2^. Scale bar, 100 μm.

Human primary hepatocytes on FBS-coated TRCDs were not confluent within 1 day after inoculation under two conditions of hepatocyte densities (1.04 and 2.08×10^5^ cells/cm^2^) (Fig. 2). After 3 days of culture, the hepatocytes showed a confluent monolayer. Hepatocytes at lower density (1.04×10^5^ cells/cm^2^) were suitable for healthy culture because little dead cells were observed.

### 1.3 Effects of layer-by-layer procedure for stable, rapid production of EHFSs

**Fig. 3:**
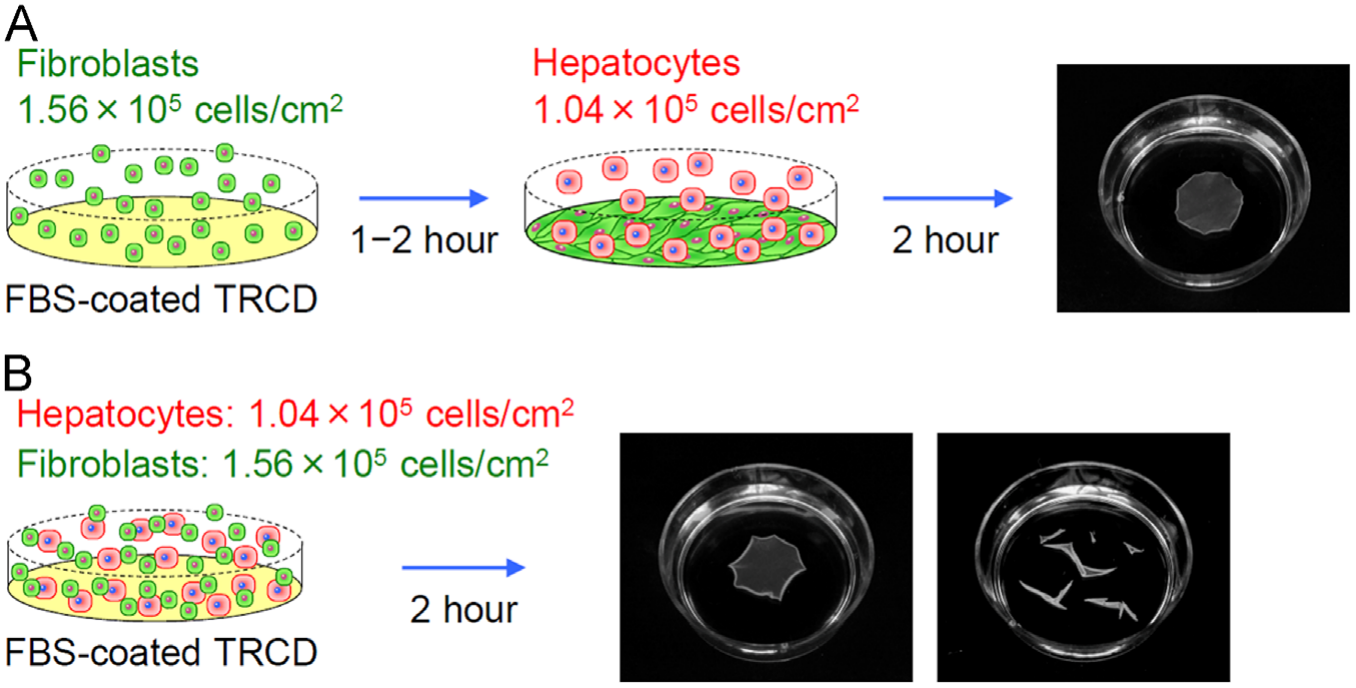
Rapid production of EHFSs: (A) layer-by-layer procedure and (B) inoculation of co-suspensions.

Human primary hepatocytes adhered onto the confluent monolayer of fibroblasts for at least 2 h after hepatocyte inoculation. EHFSs were harvested from FBS-coated TRCDs soon after the adhesion of hepatocytes by reducing the culture temperature from 37 °C to 20 °C for several minutes (Fig. 3A). Co-suspensions of hepatocytes and fibroblasts formed EHFSs, although the EHFSs were often self-detached from FBS-coated TRCDs without temperature reduction before formation of continuous cell sheet format (Fig. 3B).

## 2 Materials and methods

### 2.1 Cell preparation

Human primary hepatocytes were isolated from human liver tissues by perfusing collagenase (130 U/mL, Wako Pure Chemical, Osaka, Japan) [1]. Suspensions with >80% viable cells were used for this study. Normal human diploid fibroblast TIG-118 cells were purchased from Health Science Research Resources (JCRB0535; Osaka, Japan) [1], [2].

### 2.2 Fibroblast monolayer preparation

To determine the proper conditions for the formation of a confluent monolayer, human fibroblasts were inoculated at 1.04, 1.56, or 2.08×10^5^ cells/cm^2^ onto FBS-coated (2 h) or uncoated TRCDs. Minimum Essential Media supplemented with 10% FBS, 2 mM L-glutamine, 100 U/mL penicillin, and 100 µg/mL streptomycin was used for fibroblast culture (all from Invitrogen, Carlsbad, CA).

At 2 h of culture, the confluency of fibroblasts was measured from phase-contrast micrographs using Win ROOF Version 6.3.0 (Mitani Corp, Fukui, Japan). Data are presented as mean±standard deviation from 2 independent cell preparations.

### 2.3 Evaluation of human primary hepatocyte density

To evaluate the better density for human primary hepatocyte culture, hepatocytes were inoculated at 1.04 or 2.08×10^5^ cells/cm^2^ onto FBS-coated TRCDs. Hepato-STIM Culture Medium (BD Biosciences, San Jose, CA) supplemented with 10% FBS, 2 mM L-glutamine, 100 U/mL penicillin, and 100 µg/mL streptomycin was used for hepatocyte culture.

### 2.4 Rapid production of EHFSs

Human primary hepatocytes were plated at 1.04×10^5^ cells/cm^2^ (1.0×10^6^ cells/well) onto a confluent layer of TIG-118 fibroblasts plated 1–2 h prior at 1.56×10^5^ cells/cm^2^ (1.5×10^6^ cells/well) onto FBS-coated TRCDs (Fig. 3A). Co-suspensions of hepatocytes and fibroblasts were also inoculated onto FBS-coated TRCDs (Fig. 3B). Hepato-STIM Culture Medium supplemented with 10% FBS, 2 mM L-glutamine, 100 U/mL penicillin, and 100 µg/mL streptomycin was used for co-culture.
